# Role of Autophagy in Cancer Cell Response to Nucleolar and Endoplasmic Reticulum Stress

**DOI:** 10.3390/ijms21197334

**Published:** 2020-10-04

**Authors:** Annalisa Pecoraro, Martina Pagano, Giulia Russo, Annapina Russo

**Affiliations:** Department of Pharmacy, University of Naples “Federico II”, Via Domenico Montesano 49, 80131 Naples, Italy; annalisa.pecoraro@unina.it (A.P.); martina.pag1989@gmail.com (M.P.)

**Keywords:** nucleolar stress, autophagy, ribosomal proteins, endoplasmic reticulum stress

## Abstract

Eukaryotic cells are exposed to many internal and external stimuli that affect their fate. In particular, the exposure to some of these stimuli induces stress triggering a variety of stress responses aimed to re-establish cellular homeostasis. It is now established that the deregulation of stress response pathways plays a central role in cancer initiation and progression, allowing the adaptation of cells to an altered state in the new environment. Autophagy is a tightly regulated pathway which exerts “housekeeping” role in physiological processes. Recently, a growing amount of evidence highlighted the crucial role of autophagy in the regulation of integrated stress responses, including nucleolar and endoplasmic reticulum. In this review, we attempt to afford an overview of the complex role of nucleolar and endoplasmic reticulum stress-response mechanisms in the regulation of autophagy in cancer and cancer treatment.

## 1. Introduction

During tumorigenesis, cancer cells are strictly dependent on their higher protein demand to sustain the uncontrolled growth, resulting in an altered protein homeostasis (proteostasis) [1]. The nucleolus and the endoplasmic reticulum play both a central role in the regulation of these processes controlling the ribosome biogenesis and the folding of proteins, respectively [2,3,4]. Altered ribosome biogenesis and protein biosynthesis lead to autophagy as a general stress response [5].

Autophagy is a tightly regulated pathway in keeping cellular homeostasis and survival. This process involves dynamic degradation and recycling system of proteins and organelles, preventing the intracellular accumulation of toxic substances [6]. In most cell types, autophagy is active at basal levels, where it exerts a housekeeping role in maintaining the integrity of intracellular organelles and proteins. However, autophagy is induced as a self-protective response under several cellular stress conditions, including nutrient and growth factor deprivation, hypoxia, DNA damage, reactive oxygen species and drug treatments [6]. The autophagy starts with the assembly of protein complexes on isolated membrane to initiate the formation of an autophagosome, followed by its nucleation, elongation and maturation. Then, fusion of autophagosomes with lysosomes leads to degradation of the cargo [6]. The early stages of autophagy are characterized by the recruitment of an autophagy machinery, including ULK1 (unc51-like autophagy activating kinase 1) complex, PI3KC3-C1 (class III phosphatidylinositol 3-kinase complex I), ATG14, UVRAG (UV radiation resistance-associated gene protein) and AMBRA1 (activating molecule in BECN1-regulated autophagy protein 1), all of which are scaffolded by Beclin 1 [6]. Next, the autophagosome membrane is expanded through the association of ATG5–ATG12 complex with ATG16, forming a stable and large multimeric complex which is crucial for the stimulation and localization of LC3 I (microtubule associated protein 1 light chain 3). Then, LC3 I, interacting with phosphatidylethanolamine (PE), ATG3 and ATG7, is converted to LC3 II, which is recruited to the membrane of the autophagosome. Finally, the completed autophagosome membrane fuses with the lysosome, allowing the degradation of its cargo and recycling macromolecular precursors [6].

There are two critical nodes controlling stress-activated autophagy: mTOR (mammalian target of rapamycin) and AMPK (AMP-activated protein kinase). The former, mTOR, exists in two different complexes, mTORC1 and mTORC2, which exhibit distinct localization and function [7]. In normal condition, autophagy is maintained at basal levels by active mTOR, which phosphorylates Ser757 of ULK1 (the mammalian Atg1 orthologue), sequestering it in a complex with ATG13 and FIP200. Under stress conditions, AMPK, the main positive autophagy regulator, inhibits mTOR via phosphorylation of Raptor and, at the same time, activates ULK1 through phosphorylation of Ser317 and Ser777 with consequent induction of autophagy [7].

The role of autophagy has been deeply investigated in multiple aspects of many diseases, including cancer. In particular, in normal cells, autophagy may prevent tumor initiation by maintaining cellular and genomic integrity; however, in established tumors, autophagy allows cancer cells to survive environmental stress and is used to satisfy the high metabolic demand of these cells [7]. Furthermore, it has been recently reported that autophagy can be correlated with a high rate of malignancy, promoting tumor cell migration and invasion and inducing chemotherapy resistance [8].

In this review, we summarize the current state of knowledge of autophagy in cancer. In particular, we describe the dynamic and controversial role of autophagy in tumor progression and maintenance. We focus on the central role of autophagy in the regulation of integrated stress responses, including nucleolar and reticulum endoplasmic stress. Moreover, we try to recapitulate the multifaceted role of autophagy in cell response to cancer treatment and how its interconnection with nucleolar and ER stresses may affect the fate of cancer cells.

## 2. Dual Role of Autophagy in Cancer

Several studies have defined autophagy as a double-edge sword, representing either an oncogenic or tumor-suppressor mechanism during malignant transformation [8].

### 2.1. Autophagy in Cancer Cell Survival

In cancer biology, a huge number of studies have highlighted dynamic role of autophagy in the determination of tumor cell fate [7]. Cytoprotective effect of autophagy is strictly related to its physiological function in removing misfolded proteins, damaged organelles and ROS, thus counteracting the consequences of the genomic damage involved in cancer initiation. However, as cancer progresses, the stress-mitigating properties of autophagy are deviated by tumor cells, to satisfy the high metabolic requirements necessary for tumor survival and rapid proliferation [8].

As a tumor-suppressive mechanism, a defective autophagy has been associated with genomic instability, tumorigenesis and malignant transformation [8]. Many studies have highlighted that the depletion of the autophagy-related gene *BECN1* (encoding Beclin 1) causes the development of spontaneous tumors in mice. Allelic loss of *BECN1* was also reported in a variety of cancers, including breast, ovarian and prostate cancers [9]. In cancer-cell lines and mice models, the loss of *BECN1* has been correlated to a decrease of autophagic flux associated with an increase of cell-growth-identifying *BECN1* genes as a tumor suppressor [9]

Deficiency of *ATGs* genes has also been associated with oncogenesis. Specifically, mice with *ATG5* and *ATG7* deficiency generate liver tumors as a consequence of mitochondrial damage and oxidative stress [10]. Moreover, it has been observed that mice deficient in *ATG4* are more susceptible to develop fibrosarcoma after the exposition to chemical compounds [11]. Other studies have assigned an antitumoral function to autophagy-related genes such as *UVRAG* (UV radiation resistance-associated gene) and Bif-1 (Bax interacting factor-1), whose depletion suppresses the autophagosome formation. In particular, Bif-1 acts as a positive regulator of autophagy by interacting with Beclin-1 through UVRAG. The loss of Bif-1 strongly increases survival and proliferation in cells and promotes the development of spontaneous tumors in mice [12]. Moreover, hypoxia and metabolic stress-induced autophagy inhibit inflammation at primary sites necessary for initiation of metastasis and decrease necrosis and macrophage infiltration, resulting in a reduction of primary tumor growth [8,13].

### 2.2. Autophagy in Cancer Cell Death

Several lines of evidence identify the predominant role of autophagy as a promoter of tumor survival and growth due its ability to confer stress tolerance in cancer cells. Indeed, tumor cells are characterized by elevated metabolic and energetic demand which can be partially fulfilled by catabolic capability of autophagic process. Therefore, tumor cells are more autophagy-dependent than normal cells.

Hence, the inhibition of autophagy or reduction of autophagy genes can confer or potentiate the induction of tumor-cell death [14,15]. Previous studies suggest that the tumor microenvironment is characterized by hypoxic condition. Cancer cells are able to adapt to this condition via the activation of different stress-response pathways. Among these, HIF-1α (hypoxia-inducible factor-1 alpha) induces the expression of BNIP3/BNIP3L (atypical BH3-only proteins the Bcl-2/E1B 19 kDa-interacting protein 3) that, in turn, favors the dissociation of Bcl-2-Beclin1 complexes, activates autophagy and promotes tumor progression [16].

Yang and colleagues have detected a constitutive activation of autophagy in human pancreatic cancer cell lines and tumor specimens. They demonstrated that, after the chemotherapy treatment, the activation of autophagy may induce a state of dormancy in residual cancer cells that may promoting tumor recurrence [17].

It has been demonstrated that TRAIL (TNF-related apoptosis inducing ligand) plays a critical role in regulating the suppression of metastasis in T-cells and NK cells [18]. Notably, in TRAIL-resistant cancer cells, it has been detected an upregulation of protective autophagy correlated with an increased tumor cells viability and survival during metastasis [19]. According to all of these observations, there is a great interest in understanding how to effectively modulate autophagy to treat cancer.

## Nucleolar Stress 

The nucleolus is a sub-nuclear compartment identified as central player in the cellular-stress response [20]. Besides the well-known nucleolar canonical function as site of the ribosome biogenesis [21], several proteomic, genomic and functional studies have assigned to nucleolus novel non-canonical roles such as genome stability, cell cycle control, cellular senescence and biogenesis of ribonucleoprotein particles (RNPs) [20]. A wide range of stress stimuli may impair the nucleolar structure and/or function, leading to a complex cellular response, namely nucleolar stress, able to activate p53-dependent or -independent signaling pathways [20,22]. This condition is characterized by the disruption of nucleolar structures, causing the translocation of several nucleolar proteins from the nucleolus to the nucleoplasm, such as Nucleophosmin (NPM), nucleostemin and ribosomal proteins, such as eS7 (RPS7), uL3 (RPL3), uL18 (RPL5), uL5 (RPL11) and uL14 (RPL23).

**Figure 1:**
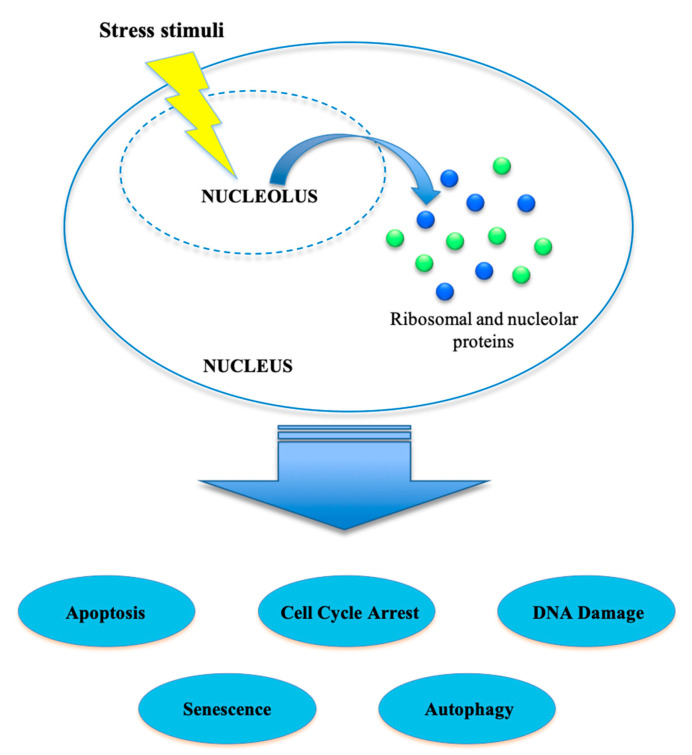
Nucleolar stress. Several stress stimuli can activate a cellular-stress-response pathway known as nucleolar stress. This condition is mediated by different ribosomal proteins and/or nucleolar proteins that are released from the nucleolus to the nucleoplasm, leading, through the activation of specific pathways, to apoptosis, cell-cycle arrest, DNA damage, senescence and/or autophagy.

The nucleolar stress pathway activation results in cell-cycle blocking, activation of apoptosis, DNA damage and senescence [20] (Figure 1).

### 3.1. Nucleolar Stress and Autophagy

Currently there is evidence that a close relationship exists between the nucleolus and autophagic process. Recently, many studies have investigated the involvement of nucleolar factors in the regulation of autophagy evidencing that nucleolar stress can be activated upstream of autophagy [5]. Thus, in the following, we discuss the main interconnections between ribosome biogenesis machinery, nucleolus and autophagy.

Different studies suggest the implication of the nucleolar-resident RNA Polymerase I (Pol I), the main actor in ribosomal RNA precursor transcription, in the nucleolus-mediated autophagy [5]. It is well-known that the specific inhibition of Pol I leads to nucleolar disruption and consequently to the translocation of several nucleolar proteins from the nucleolus to the nucleoplasm. Katagiri and colleagues have shown that NPM plays a key role in the induction of specialized form of nucleolus-mediated autophagy via mechanisms different from those involved in canonical autophagy [23].

Nucleolar disruption stabilizes p53 and increases its activity leading to the expression of multiple p53 target genes [24]. Beside its function on cell-cycle regulation and apoptosis, p53 can be considered as either an inducer or an inhibitor of autophagy, depending on its subcellular localization [25]. Under physiological condition, cytosolic p53 protein exerts a negative regulation on autophagy through a transcription-independent mechanism. This inhibitory effect involves the AMPK–mTOR signaling pathway via inhibition of AMPK and, consequently, activation of mTOR [26].

Nevertheless, upon cellular stress, p53 protein translocates to the nucleoplasm, where it triggers pro-autophagic functions [25]. Nuclear p53 acts at different levels: It induces the expression of many autophagy-related genes, such as ATGs, ULK1 and Parkin; it inhibits the mTOR pathway via activation of AMPK or by increasing PTEN expression; and it induces Beclin1 through BAX and Bcl-2 regulation [27]. Reduced expression or loss of the human tumor-suppressor PICT-1 (protein interacting with carboxyl terminus 1) is correlated with high malignant progression of several cancers by supporting anchorage-independent cancer cell growth and reducing cellular pro-apoptotic response [28]. Other studies demonstrated that PICT-1 overexpression suppresses anchorage-independent cancer cell growth and activates mitochondria-independent cell death [29,30]. Recently, it has been elucidated the involvement of PICT-1 in the regulation of ribosome biogenesis. Chen and colleagues showed that PICT-1 binds rDNA genetic loci and its overexpression inhibits the activation of UBF1 (upstream binding transcription factor) and the recruitment of RNA Pol I to rDNA with consequent reduction of rDNA transcription. Furthermore, the authors demonstrated that PICT-1 overexpression potently induces pro-death autophagy in cancer cells, and these effects depend on its nucleolar localization. Of note, the increased levels of PICT-1 did not affect the integrity of nucleolar structure and p53 expression levels, suggesting that the dysregulation of ribosomal biogenesis is the main cause of PICT-1-induced autophagy. Overall, these data highlight PICT-1′s tumor-suppressor function and identify this nucleolar factor as a potent regulator of nucleolus-mediated autophagy [30].

### 3.2. Ribosomal Proteins and Autophagy

It has been shown that autophagy induction is correlated to the alteration of the expression of some ribosomal proteins. For example, depletion of RPLP proteins, including RPLP0, RPLP1, and RPLP2, induced autophagy in breast and ovarian cancer cell lines [31]. Disruption of the ribosomal P complex triggers stress-mediated autophagy. Immunohistochemistry analysis of breast tissue microarray revealed that the expression of ribosomal protein S27-like (RPS27L), an evolutionarily conserved ribosomal protein of 40S small subunit, was found to be lower in breast tumors than in normal breast tissues, suggesting that it may play a role in breast tumorigenesis. These findings are in accordance with data obtained in colorectal cancer, where the low expression of RPS27L in either feces or cancer tissues is related with a worse patient prognosis. Silencing of RPS27L significantly induced protective autophagy in breast cancer cells through the inactivation of mTORC1 [32]. Specifically, RPS27L depletion is associated to a reduction of β-TrCP half-life with consequent accumulation of DEPTOR, an inhibitor of mTOR. Altogether, these findings identified a new autophagy regulator axis in RPS27L-β-TrCP-DEPTOR-mTORC1 [32].

**Figure 2:**
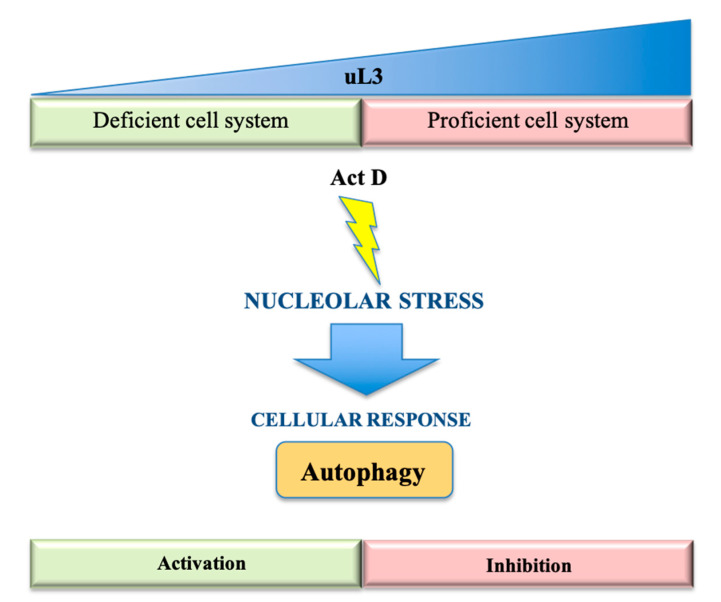
Effect of uL3 (RPL3) status on autophagy. The nucleolar stress response upon Act D treatment depends on uL3 status. Reduced uL3 levels cause a cellular response employing autophagy induction, while increased uL3 amounts inhibit this process.

Recent findings obtained by our research group strongly suggest that nucleolar stress and autophagy are tightly coupled in colon cancer, evidencing the ability of ribosomal protein uL3 to act as a repressor of autophagy [33]. We have previously demonstrated that uL3 is a key mediator of nucleolar stress induced by several chemotherapeutic drugs, including 5-fluorouracil (5-FU) [34,35,36], Oxaliplatinum (OHP) [37,38], Actinomycin D (Act D) [39,40] and Niclosamide, in p53-mutated lung and p53-deleted colon cancer cells [41,42]. Specifically, we identified a new nucleolar stress pathway activated upon cell treatment with chemotherapeutic drugs that is p53-independent and uL3-dependent [22,43,44]. Transcriptome analysis of genes and pathways that are differentially expressed in colon cancer cells devoid of p53, in the presence or in absence of uL3, and in condition of nucleolar stress activated by Act D, revealed that uL3 deficient colon cancer cells showed the upregulation of pathways related to autophagy activation. Among these, the most relevant is mTORC1. Overall, our data demonstrated that uL3 depletion may enhance the resistance of colon cancer cells to drug treatment through autophagy induction, whereas the restoration of uL3 drives colon cancer cells’ death by autophagy inhibition [33] (Figure 2).

## 4. Endoplasmic Reticulum Stress 

Endoplasmic reticulum is a specialized organelle responsible for folding and post-translational processing of membrane-bound and secreted proteins, lipid synthesis, degradation of glycogen, detoxification, and Ca^2+^ storage and release. The quality-control systems of the endoplasmic reticulum (ER) selectively regulate tracking of the well-folded proteins and target the misfolded ones for proteolysis. Different conditions can alter the ER homeostasis, such as nutrient deprivation, hypoxia, acidosis, drug-induced toxicity and irradiation, resulting in the accumulation of the misfolded and unfolded proteins within the lumen of the ER and, consequently, contributing to ER stress development [45].

In order to counteract the occurring damage, cells have evolved a conserved signal transduction pathway called unfolded protein response (UPR). Firstly, the UPR attempts to re-establish ER homeostasis by slowing down ongoing protein synthesis and increasing the folding capacity of the ER. The unfolded proteins can still achieve correct conformation through further processing by ER chaperones such as calreticulin, calnexin and ER resident protein 57 (ERp57); can target the so-called ER-associated degradation (ERAD); or can undergo autophagy. However, if the initial adaptive responses fail to restore proteostasis, UPR signaling persists evolving in “Terminal UPR” signals. During the activation of this alternative signaling program, the proapoptotic proteins belonging to Bcl-2 family (BH3-only proteins) become activated culminating in programmed cell death [46].

The UPR signal is controlled by three different ER transmembrane sensors: inositol requiring enzyme1α (IRE1α), protein kinase RNA(PKR)-like ER kinase (PERK) and activating transcription factor 6 (ATF6) [46]. Under physiological conditions, the main represented ER-resident chaperone, known as glucose-regulated protein 78 (GRP78) or binding immunoglobulin protein (b), is bound to the ER luminal domain of the three sensors, to maintain them in an inactive state. The disruption of protein homeostasis during ER-stress leads to the detachment of BiP from the three ER-sensors to cooperate in protein folding. Upon dissociation of BiP binding, IRE1 and PERK form homodimers or oligomers, whereas ATF6 translocates to the Golgi apparatus, activating their downstream pathways [47].

**Figure 3:**
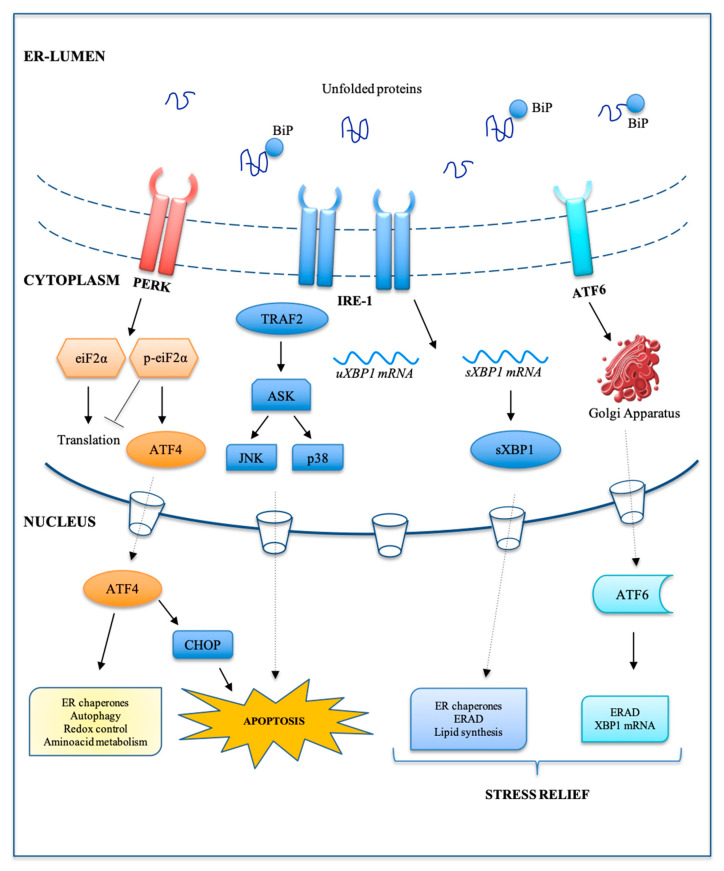
Schematic representation of unfolded protein response (UPR) response. The UPR signal is driven by three different endoplasmic reticulum (ER) transmembrane sensors: protein kinase RNA(PKR)-like ER kinase (PERK), inositol requiring enzyme1α (IRE-1) and activating transcription factor 6 (ATF6). Upon unfolded protein accumulation, binding immunoglobulin protein (BiP) dissociates from IRE1 and PERK, whereas ATF6 translocates to the Golgi apparatus, to be activated. The activation of these three branches leads to the activation of their downstream pathways.

During tumorigenesis, the three arms of the UPR are highly active and shift the balance between cell survival and cell death, deciding the fate of the cancer cells. If the different UPR-mediated mechanisms fail to counteract ER stress, apoptotic pathways can be activated [47]: PERK/eIF2α-dependent induction of CCAAT-enhancer-binding protein homologous protein (CHOP); IRE1-mediated activation of TRAF2, which stimulates the ASK1/JNK kinase cascade, and Bax/Bcl2-regulated Ca^2+^ release from the ER. CHOP/GADD153 (growth arrest/DNA damage) plays a convergent role in the UPR and has been identified as one of the most important mediators of ER stress-induced apoptosis [47,48] (Figure 3).

ER stress exerts both anti-tumorigenic and pro-tumorigenic effects in cancer, depending on the severity of induced ER stress. Persistent and prolonged ER stress may switch the cytoprotective functions of autophagy to cell-death-promoting mechanism [47].

In fact, although prolonged UPR activation induces apoptotic signaling, cancer cells can exploit the UPR as an adaptive mechanism to support survival and chemo-resistance of tumor cells. A strategy to improve the cancer therapy can be the targeting of alternative pathways connected to UPR response. UPR may increase the autophagy process, but if this process gets to a point of no return, it will promote cell death. Therefore, the treatment of tumor cells with ER-induced drugs might cause the induction or the breakdown of pro-death branch of the UPR adaptive system.

### 4.1. Endoplasmic Reticulum Stress and Autophagy

A huge number of studies have highlighted that ER stress and autophagy are strictly interconnected. The first evidence of this link was reported by Bernales and colleagues in 2006. They observed that, under ER stress response, ER membranes become selectively sequestered in autophagosome-like structures [49]. The three canonical branches of the UPR regulate the autophagy in distinctive manners during the ER stress. For instance, various Ca^2+^ mobilizing agents, such as ionomycin, ATP (via purinergic receptors) and thapsigargin (an irreversible inhibitor of the ER Ca^2+^ATPase), inhibit the activity of mTOR, a negative regulator of autophagy, and induce massive accumulation of autophagosomes in Beclin-1 and Atg7-dependent manner [50]. In this regard, it has been proposed that Ca^2+^ release from the ER through the IP3R channel induces the phosphorylation of CaMKKβ and activates AMPK, which, in turn, inhibits mTOR inactivating ULK1 complex, thus inducing autophagy [50,51]. In addition, Ogata and colleagues have investigated the regulation of ER stress-induced autophagy by IRE1α. Specifically, the interaction between IRE1α and TRAF2 triggered JNK activation [52], which, in turn, caused the release of Beclin-1 via phosphorylation of Bcl-2, allowing autophagy to go on [53]. On the other hand, Kouroku and colleagues showed that the PERK/elF2α signaling pathway induced autophagy through ATF4-driven transcriptional regulation as ATG12, ATG16L and DDIT3 [54]. Similarly, CHOP can be considered an inducer of autophagy via the activation of TRIBB3, which inhibits the AKT/mTOR pathway signaling [55]. Recently, it has been demonstrated that sXBP-1 triggers autophagy through the transcriptional activation of Beclin-1 and consequent decrease of FoxO1 activity [56]. Moreover, the inhibition of AKT/mTOR pathway can be mediated also by ATF6 arm of UPR branch [57].

### 4.2. Ribosomal Proteins and Endoplasmic Reticulum Stress

It is well-known that, under ER stress condition, PERK-dependent eIF2 phosphorylation can inhibit Pol I activity interfering with the formation of the 43S translation initiation complex and thus attenuating protein translation. Phosphorylated eIF2α, through the reduction of the formation of mature polysomes, causes an increase of free r-proteins [58,59]. Several reports indicate that r-proteins, in addition to their role as components of translation machinery, exert a variety of extra-ribosomal functions involved in the regulation of different cellular process [20]. Among these, there is new evidence about the interconnection between UPR and r-proteins.

It has been demonstrated that eL22 (Rpl22) loss exacerbates ER stress and strongly activates two of the three ER stress-signaling pathways, PERK and IRE1α [60]. Zhang et al. have shown that UPR induction promotes the interaction between the r-proteins (uL18/rpL5, uL5/rpL11 and uL14/rpL23) and Hdm2 in PERK-dependent manner. This interaction results in the inhibition of Hdm2-mediated ubiquitination and degradation of p53, leading to cell cycle arrest. Therefore, Hdm2/p53 signaling mediates the cross-talk between ribosome biogenesis and cell cycle [61]. These data strongly suggest that the perturbation of the ribosome biogenesis plays an essential role in coupling the UPR to cell cycle regulation. 

In the last decade, our research group has deeply investigated the extra-ribosomal roles of r-protein uL3 [62,63]. More recently, our data have shown the activation of UPR pathway in colon cancer cells devoid of p53 and stably silenced of uL3 by RNA seq analysis, suggesting a role of uL3 in the regulation of UPR [33]. Moreover, it has been demonstrated that the reduction of uL3 expression levels is associated to drug-resistance phenotype in colon cancer cells; thus, we can speculate that the activation of UPR in absence of uL3 could contribute to sustain this phenotype [33].

According to the dual role of UPR in cancer cells, some evidence indicates that stressful conditions such as those occurring during cancer activate adaptive responses that are controlled by the NF-κB signaling pathway, as well as by UPR.

NF-κB activation may represent another match point between ER and nucleolus, since it can be not only controlled by all three UPR branches but also targeted by several r-proteins [64,65].

The role of r-proteins in the regulation of this process is controversial. In fact, uL3 prevents the degradation of IκB upon 5-FU treatment, thus repressing NF-κB activity, while ribosomal protein uS3 promotes NF-κB activity by interacting with NF-κB complexes in the nucleus [34,66].

Therefore, understanding the molecular mechanism by which ER and nucleolus are interconnected represents an emerging area of investigation and will provide an important tool for the development of new targeted therapeutic approaches in cancer.

## 5. Autophagy in Cell Response to Cancer Treatment

Given the dual role of autophagy in cancer, the inhibition, but also the induction, of autophagy may represent a potential target in cancer therapy. Several studies reported that therapeutic induction of autophagic cell death, also known as type 2 cell death, is a critical process to trigger tumor cell elimination. 

### 5.1. Drugs Modulating Autophagy

Temozolomide (TMZ) is the first pro-autophagic cytotoxic drug used to enhance apoptosis in resistant cancer cells and was approved for use in glioblastoma multiforme (GBM) [67]. TMZ is a DNA-alkylating agent that induces the formation of *O*-6-methylguanine in DNA, resulting in DNA damage by mispairing with thymine. It has been demonstrated that TMZ is able to induce autophagy cell death by the recruitment of LC3 to autophagosomal membranes. Interestingly, the pharmacological inhibition of autophagy by 3-MA (3-methyladenine) strongly decrease TMZ cytotoxicity providing evidence that the antitumor effect of this drug is mediated by autophagy [68].

Metformin (*N*′,*N*′-dimethylbiguanide) is an oral hypoglycemic agent extracted from the herb *Galega officinalis* and is widely used for treating type II diabetes. In the last decades, Metformin has received increased attention for its potential anticancer effects [69]. Indeed, several studies have been demonstrated that Metformin is able to inhibit cell growth in different types of cancer, including breast, lung, colorectal, pancreatic, head and neck, and prostate cancer [70,71,72]. Moreover, it has been reported that Metformin is able to induce autophagy by AMPK activation and consequent inhibition of mTOR [69]. In particular, Feng Y. and colleagues have showed that Metformin induces autophagy and downregulates STAT3 signaling, causing the inhibition of the esophageal squamous cell carcinoma cell growth [73]. In addition, a study on human multiple myeloma cells demonstrated that Metformin inhibited cell growth, triggering autophagy and G0/G1 cell cycle arrest. These effects are correlated to the upregulation of AMPK and the downregulation of mTOR observed upon Metformin treatment [74]. Recently, De Santi and colleagues have showed that Metformin inhibited cancer initiation and progression through autophagy-related cell death [75]. Notably, the inhibition of autophagy by wortmannin or ATG7 silencing reduces Metformin anticancer effects [75].

Bortezomib is a novel first-in-class proteasome inhibitor. It is an inhibitor of the 26S proteasome and is currently an FDA-approved drug for the treatment of multiple myeloma [76]. The anticancer activities of Bortezomib have been deeply investigated, providing evidence of its effectiveness in various human cancers as colon, prostate, breast and ovarian cancer [77,78]. Besides its role as proteasome inhibitor, it has been observed that Bortezomib specifically inhibits NF-κB pathway and strongly sensitizes cancer cells to chemotherapy-mediated apoptosis [76]. Furthermore, several studies have investigated its possible involvement in the modulation of autophagy in breast cancer, melanoma, head and neck cancer, hepatocellular carcinoma and prostate cancer. In ovarian cancer and other solid tumors, this compound may inhibit the autophagic process at the autophagolysomal stage by inducing ERK phosphorylation. Interestingly, Bortezomib enhances the anticancer effects of cisplatin via inhibition of cisplatin-induced autophagy [79]. In contrast, Li and colleagues have demonstrated a pro-autophagic role of Bortezomib in head and neck cancer, although the mechanism is not yet completely clarified. In this study, they demonstrated that Bortezomib strongly enhance autophagy via JNK activation and phosphorylation of Bcl-2 [80].

### 5.2. BH3 (Bcl-2 Homology 3) Mimetics

BH3 mimetics are molecules that mimic interactions of BH3-only proteins, a subset of pro-apoptotic proteins belonging to Bcl-2 family [81]. Different studies have reported that autophagy-mediated cell death caused by BH3 mimetics can occur through the recruitment of Beclin-1 from Bcl2 and Bcl-XL inhibition [82].

Gossypol is a natural polyphenolic compound derived from cottonseed extract; it is a BH3 mimetic that shows proapoptotic effects in several in vitro and in vivo models [83,84,85]. It is composed by two enantiomers, (+)-gossypol and (−)-gossypol, with the latter results more potent to suppress tumor growth. Gossypol acts as pan-BCL-2 inhibitor by inactivating the pro-survival members of BCL-2 family as BCL-xL, MCL-1, and BCL-w. Specifically, this natural compound binds BH3 hydrophobic grooves of the pro-survival proteins and activates both apoptosis and autophagy [86,87].

It has been demonstrated that (−)-gossypol triggers autophagic cell death in combination with TMZ in apoptosis-resistant malignant glioma cells [88]. Notably, the inhibition of autophagy by silencing of BECN1 or ATG5 strongly decreased cell death caused by (−)-gossypol alone or in combination with TMZ [88]. 

Another BH3 mimetic whose antiproliferative activity seems to be mediated by autophagy cancer cell death is Obatoclax. It has been shown that, in genetic silencing of autophagy-related genes, such as BECN1, ATG5 and ATG7, Obatoclax failed to cause cell death, evidencing their importance in Obatoclax-mediated antiproliferative effects. Furthermore, it has been demonstrated that Obatoclax enhances the conversion of LC3 through the Beclin-dependent mechanism in B-cell lymphoma [89]. This compound exerts also anti-leukemic activity by triggering autophagy in pediatric acute lymphoblastic leukemia [90].

### 5.3. Cannabinoids

Tetrahydrocannabinol (THC) represents the main active component of cannabinoids. In glioblastoma and hepatocellular carcinoma, this compound acts as a stimulus for autophagy cell death [91,92]. Specifically, when ULK1, ATG5 or Ambra-1 are silenced, the glioblastoma cells become more resistant to THC treatment [91]. Salazar and colleagues have identified autophagy as a process upstream of apoptosis in cannabinoid-induced human and mouse cancer cell death. They showed that THC induced ceramide accumulation and eIF2α (eukaryotic translation initiation factor 2α) phosphorylation, leading to the activation of endoplasmic reticulum (ER) stress response that promoted autophagy via TRB3-dependent (Tribbles homolog 3–dependent) inhibition of the Akt/mTORC1 axis. Altogether, these findings delineate the mechanism underlining THC pro-autophagic death, thereby suggesting cannabinoid administration for therapeutic purpose [91].

JWH-015 is a cannabinoid receptor 2-selective agonist involved in autophagy cell death in hepatocarcinoma cells. The activation of autophagy employed two different mechanisms, the inhibition of Akt/mTOR axis and the activation of AMPK signaling, both in vitro and in vivo. Silencing of ATG5 or pharmacological inhibition of autophagy by using 3-MA prevented the decrease in tumor growth evoked by JWH-015. Altogether, these findings strongly hold up the antitumor action of cannabinoids via autophagy activation in hepatocellular carcinoma [92,93].

### 5.4. Epigenetic Modifiers

An example of the epigenetic control of autophagy has been demonstrated through acetylation of histones. Histone deacetylase inhibitors show anticancer effects by inducing autophagic cell death in chondrosarcoma cell lines. Suberoylanilide hydroxamic acid (SAHA) has been reported to induce autophagy associated with ultrastructural changes in autophagosome formation and enhancement of lipidation of LC3 [94]. Interestingly, pharmacological inhibition of autophagy by 3-MA significantly abrogated the effects of SAHA treatment [94].

### 5.5. mTOR Inhibitors

Given the central role of mTOR signaling in the regulation of autophagy, the inhibition of this mechanism in cancer cells has been deeply investigated over the last decade. Rapamycin (RAPA) is a special prophylactic for mTOR which binds FKBP12 (fk506-binding protein 12 kDa) to form a molecular complex that inhibits mTOR activity. Furthermore, the induction of autophagy associated to mTOR inhibition may mediate some effects on tumor development [95]. Rapamycin has been shown to inhibit proliferation and induce autophagic cell death in murine sarcoma, neuroblastoma, lung cancer and osteosarcoma [96,97,98,99]. In addition, Everolimus (or RAD001), a rapamycin analogue, was approved by FDA for use in different types of tumors, including advanced renal cell carcinoma, advanced pancreatic neuroendocrine tumors, renal angiomyolipoma and HER2-negative breast cancer. This drug has shown to induce cell cycle arrest via autophagy-mediated degradation of Cyclin D1 in breast cancer cells [100]. In addition, Everolimus contributes to the development of drug resistance by promoting autophagy in aromatase-inhibitor-resistant breast cancer cells [101].

Other inhibitors of mTOR are compounds able to compete with ATP, hampering phosphorylation of its target proteins, causing a more efficient inhibition of mTOR [102]. An example is represented by AZD8055 that inhibits both mTOR complexes. It has been demonstrated that AZD8055 suppresses tumor growth [103] and induces autophagy-mediated cell death in hepatocellular carcinoma cell lines [104]. In addition, AZD8055 inhibits cancer cell proliferation through induction of apoptotic death and cell cycle arrest [105].

Altogether, these findings suggest that mTOR inhibitors may act through different mechanisms to promote cancer cell death in a cellular-context-dependent manner.

### 5.6. ATG Inhibitors

It is well established that the enhancement of autophagic process is often related to cancer cell survival and proliferation [8]. Thus, the employment of autophagy inhibitors could represent a useful strategy in cancer therapy. In this light, the silencing of ATG genes or pharmacological inhibition of autophagy can effectively promote tumor cell death induced by different anticancer drugs in preclinical models [106]. In apoptosis-defective leukemic and colon cancer cell lines, inhibition of autophagy was shown to sensitize resistant cells to TRAIL-mediated apoptosis [19]. Furthermore, inhibition of autophagy enhanced apoptosis induction by cetuximab, an antibody against EGFR [107].

### 5.7. Lysosome Inhibitors

The last step in the autophagic process is the fusion of autophagosomes with lysosomes in order to degrade the autophagosome content [6].

Current efforts are focused on inhibiting the lysosomes by using CQ (chloroquine) or the related HCQ (hydroxychloroquine). Both of them are used for the treatment of different disease such as malaria and, more recently, cancer. At neutral pH, CQ/HCQ is unprotonated and can freely diffuse through cell membranes and enter into organelles, such as lysosomes, where acid environment causes their protonation and consequently increases lysosomal pH. Once CQ/HCQ is protonated, it is trapped in the lysosomes, causing an increase of its volume and hindering the activity of degradative enzymes. Consequently, this compound, by inhibiting lysosomal acidification, causes the blockage of autophagosome fusion and degradation, preventing, in this way, autophagy [108]. Of note, CQ and HCQ are the only available autophagy inhibitors approved for clinical use. It has been demonstrated that CQ and HCQ synergize with multiple drugs, such as 5-FU [109], cisplatin [110] and temozolomide [111], potentiating their cytotoxic effects. Furthermore, combination treatment with CQ and trastuzumab completely blocks tumor growth in HER2-positive breast cancer tumor xenograft characterized by resistance to trastuzumab [112]. Autophagy inhibition by CQ is also connected with the accumulation of proteins in the cytoplasm, which, in turn, leads to the activation of ER stress and results in further cytotoxic effects [113]. Many preclinical studies have confirmed that the effective autophagy inhibition can be achieved with CQ and HCQ, providing further evidence of therapeutic application in cancer. However, it is still not clear if the cytotoxic effects of these agents in cancer cells are specifically connected with autophagy inhibition and not with another effect on lysosomes. Notably, cancer cells that exhibit an increased autophagy flux are sensitive to, and could die in response to treatment with, lysosomotropic agents [114].

Although HCQ shows a wide tissue distribution, determining a high intratumoral concentration, it has been reported that CQ or HCQ may not be powerful enough to exert therapeutic efficacy in tumors at tolerable doses. Hence, several derivatives of CQ or HCQ have been developed to enhance their activity. Lys05, a bivalent aminoquinoline analog of HCQ, is water soluble and accumulates more readily within the lysosome, causing a strong increase of lysosomal pH with consequent inhibition of autophagy [115]. Notably, Lys05 triggers cytotoxicity with a higher potency than HCQ in tumor cell lines [116].

### 5.8. Natural Compounds

Recently, the scientific interest has been focused on the research of new natural compounds with potential anticancer activity in order to overcome the problem of chemotherapy toxicity. To date, a wide range of natural molecules obtained from plants, marine organisms and microorganisms are able to arrest cancer cell proliferation by autophagy inhibition or induction. 

Artemisinin (ART) is a natural molecule derived from the medicinal herb *Artemisia annua*. It has been demonstrated that Artemisinin treatment selectively triggers cell death via autophagy modulation [117]. However, Artemisinin’s use as anticancer drug is limited by its low potency. Thus, Ganguli and colleagues have observed that the combined treatment with CQ cause an increase of Artemisinin antiproliferative action in lung cancer cells [118].

Dihydroartemisinin (DHA), the main active metabolite of Artemisinin, triggers autophagy in many cancer cell lines, employing the suppression of NF-kB activation and the accumulation of ROS [118,119]. Recently, it has been investigated the synergistic combination of DHA with cisplatin [120] and TMZ [121], providing new evidence on the ability of this natural compound to enhance the cancer cells’ sensitivity to chemotherapy treatment.

Curcumin (CUR) is a polyphenolic compound derived from the plant *Curcuma longa*, widely employed as food additive, as well as an effective medicine for various disorders. Lee et colleagues have demonstrated that Curcumin-induced autophagy involved ROS production, Beclin-1 upregulation and, consequently, LC3-II accumulation, causing colon cancer cell death [122]. A recent study has showed that Curcumin treatment also induced the activation of TFEB (transcription factor EB), a master nuclear transcription factor involved in autophagy and lysosome biogenesis regulation [123]. The activation of TFEB induces autophagy by mTOR axis inhibition, leading to greater autophagic cell death [123]. In addition, the efficacy of the combined treatment with Curcumin and TMZ is strongly enhanced by the inhibition of protective autophagy [124]. 

Resveratrol belongs to polyphenols’ stilbenoids group. It has been deeply investigated for its anticancer properties. Several studies have highlighted the interconnection between autophagy and Resveratrol in cancer cells. In particular, Resveratrol treatment induces autophagy-mediated cell death in breast cancer stem cells, by suppressing the Wnt/β-catenin pathway [125]. Fan and colleagues have demonstrated that the pro-apoptotic effect of Resveratrol was due to increased levels of LC3-II protein and autophagosomes in leukemia cells [126]. In addition, Resveratrol treatment causes a reduction of SOCE (store-operated calcium entry), a cellular mechanism that ensures the balance of cellular calcium and induces autophagy. Altered SOCE influx triggers ER stress response, which further increases pro-death autophagy via inhibition of AKT/mTOR pathway [127].

In the following table (Table 1), we provide a general classification of current available compounds targeting autophagy machinery.

### 5.9. Linking Nucleolar and ER Stress to Autophagy: Future Perspectives in Cancer Therapy 

New efforts in cancer therapy are currently directed to simultaneous regulation of multiple cellular pathways by a single or combining agents.

Act D is a potent intercalating agent and was the first antibiotic exerting anticancer activity. Typically, low doses of Act D cause a specific inhibition of RNA pol I driven transcription, impairing ribosome biogenesis with consequent nucleolar stress induction [39]. Notably, Cortes and colleagues have demonstrated that Act D treatment induced pro-death autophagy in p53-deficient neuroblastoma cells [128]. Besides its antitumoral activity as single agent, they have also observed that Act D had synergistic effect with SAHA, a well-known autophagy inducer [128].

CX-5461 is a small compound belonging to the next generation RNA polymerase inhibitors. The high antitumor potency of CX-5461 has been evaluated and confirmed in several studies, both in vitro and in vivo [129]. Recently, Duo and colleagues developed a CX-5461-loaded nanoplatform, which accumulates in nucleoli. The anticancer properties of this nanoplatform were due to the inhibition of rRNA transcription and activation of pro-death autophagy. It is of interest that the CX-5461-loaded nanoplatform did not exhibit any relevant toxicity, identifying it as a safe and potentially nucleolus-targeting anticancer drug [130].

In cancer cells, it has been shown that a wide array of antitumoral agents may stimulate ER stress and activation of UPR along with autophagy. Among the principal ER stress inducers, there are tunicamycin, thapsigargin and brefeldin A. Although these compounds act by different mechanisms, they finally lead to the accumulation of unfolded proteins in the ER, resulting in the activation of UPR. Notably, recent studies have demonstrated that these classical ER stress inducers may activate also the autophagic flux. The autophagy activation mitigates ER stress and has a protective role for cancer cell survival [131]. Thus, a combined therapy with autophagy inhibitors could be useful approach for certain types of cancer in which the main cause is represented by ER stress response. 

## 6. Conclusions

Disturbance of autophagy was found to be one of the possible causes of tumor formation and progression. Reduced autophagy can contribute to tumor progression, whereas increased autophagy may be a mechanism for tumor survival under hypoxic-, metabolic- or therapeutic-stress conditions. A better understanding of the stress pathways as nucleolar and endoplasmic reticulum stress and autophagy could open novel avenues for investigating specific and effective pharmacologic targets for new drug development and therapeutic approaches for cancer treatment.

## Figures and Tables

**Table 1:** Classification of drugs targeting autophagy in cancer therapy.

Classification	Drugs	Mechanism of Action
***Autophagy modulators***	Temozolomide (TMZ)	Induces autophagy by LC3 recruitment to autophagosomal membranes [68]
	Rapamycin (RAPA)	Induce autophagy by TOR inhibition [95,96,97,98,99,100,101,102,103,104,105]
	Everolimus	
	AZD8055	
	Metformin	Induces autophagy by AMPK activation [69,74]
	Bortezomib	Inhibits autophagy by inducing ERK phosphorylation and synergizes with cisplatin [79]
***BH3*** ***(Bcl-2 homology 3) mimetics***	Gossypol	Induce autophagy by Beclin1-dependent mechanism [86,87,89,90]
	Obatoclax	
***Cannabinoids and cannabinoid agonists***	Tetrahydrocannabinol (THC)	Induces autophagy via TRB3-dependent inhibition of AKT/mTORC1 pathway [91]
	JWH-015	Induces autophagy via the inhibition of AKT/mTOR axis and the activation of AMPK signaling [92,93]
***Epigenetic modifiers***	Suberoylanilide hydroxamic acid (SAHA)	Induces autophagy the enhancement of LC3 lipidation [94]
***Lysosome inhibitors***	Chloroquine (CQ)	Inhibit autophagy by the blockage of autophagosome fusion and degradation [108,113,115]
	Hydroxychloroquine (HCQ)	
	Lys05	
***Natural compounds***	Artemisinin (ART) Dihydroartemisinin (DHA)	Induce autophagy by NF-kB inhibition and ROS accumulation [117,118]
	Curcumin (CUR)	Induces autophagy by Beclin-1 upregulation, LC3II accumulation [122] and activation on TFEB [123]
	Resveratrol	Induces autophagy by Wnt/β-catenin pathway suppression [125] and reduction of SOCE [127]
